# *In Vitro* Proliferation of Porcine Pancreatic Islet Cells for *β*-Cell Therapy Applications

**DOI:** 10.1155/2016/5807876

**Published:** 2016-12-06

**Authors:** Guoguang Niu, John P. McQuilling, Yu Zhou, Emmanuel C. Opara, Giuseppe Orlando, Shay Soker

**Affiliations:** ^1^Wake Forest Institute for Regenerative Medicine, Wake Forest Baptist Medical Center, Medical Center Boulevard, Winston-Salem, NC 27157, USA; ^2^Virginia Tech, Wake Forest University School of Biomedical Engineering and Sciences, 320 ICTAS, Stanger St., Virginia Tech, Blacksburg, VA 24060, USA

## Abstract

*β*-Cell replacement through transplantation is the only curative treatment to establish a long-term stable euglycemia in diabetic patients. Owing to the shortage of donor tissue, attempts are being made to develop alternative sources of insulin-secreting cells. Stem cells differentiation and reprograming as well as isolating pancreatic progenitors from different sources are some examples; however, no approach has yet yielded a clinically relevant solution. Dissociated islet cells that are cultured in cell numbers by* in vitro* proliferation provide a promising platform for redifferentiation towards *β*-cells phenotype. In this study, we cultured islet-derived cells* in vitro* and examined the expression of *β*-cell genes during the proliferation. Islets were isolated from porcine pancreases and enzymatically digested to dissociate the component cells. The cells proliferated well in tissue culture plates and were subcultured for no more than 5 passages. Only 10% of insulin expression, as measured by PCR, was preserved in each passage. High glucose media enhanced insulin expression by about 4–18 fold, suggesting a glucose-dependent effect in the proliferated islet-derived cells. The islet-derived cells also expressed other pancreatic genes such as Pdx1, NeuroD, glucagon, and somatostatin. Taken together, these results indicate that pancreatic islet-derived cells, proliferated* in vitro*, retained the expression capacity for key pancreatic genes, thus suggesting that the cells may be redifferentiated into insulin-secreting *β*-like cells.

## 1. Introduction

Diabetes mellitus is a complex chronic disease that affects more than 380 million people worldwide and consumed at least USD 612 billion dollars in health expenditure in 2014, according to the International Diabetes Federation (http://www.idf.org/). Around 10% of all cases are type 1 diabetes (T1D), which results from an autoimmune reaction where the body's defense system attacks and destroys insulin-producing *β*-cells in pancreatic islets. However, most patients have type 2 diabetes (T2D), which results from the peripheral tissue insulin resistance and *β*-cell dysfunction [1, 2]. Owing to the difficulty in maintaining glucose homeostasis, diabetic patients have increased risks of developing a number of serious health problems in the presence of consistently high blood glucose levels. These comorbidities include cardiovascular disease [3, 4], blindness [5], kidney failure, and lower limb amputation [6, 7]. Islet transplantation is an effective strategy to potentially cure T1D and eliminate the need for daily insulin injections [8]. Patients transplanted with cadaveric human islets can remain insulin independent for 5 years or even longer via this therapy [1, 9, 10]. However, this approach is greatly limited due to the shortage of donor islets. During islet isolation, a significant proportion of islets is lost and 2-3 human donor organs per recipient are required to obtain the desired islet mass, thus exacerbating the current shortage of donated pancreases and denying millions of patients the benefit of transplantation [2]. With recent progress in the fields of developmental biology and regenerative medicine the idea of generating an unlimited supply of *β*-cells* in vitro* has emerged as a potential source to extend islet transplantation to millions of patients afflicted with T1D.

There are many different cell sources, from which *β*-cells can be generated [11, 12]. Human *β*-cells have a considerable low rate of replication, and it has been shown that purified human *β*-cells failed to replicate* in vitro* regardless of the substratum or growth factors used [13]. Lineage-tracing experiments have demonstrated that loss of *β*-cell mass was due to *β*-cell dedifferentiation and that the dedifferentiated *β*-cells reverted to progenitor-like cells [14]. Indeed, some groups have suggested that *β*-cells of cultured islets could be dedifferentiated and expanded in numbers during* in vitro* proliferation and then be directed to redifferentiate back towards *β*-cells [11, 15]. Gershengorn et al. have also indicated that fibroblast-like cells derived from adult human islets proliferated* in vitro* and underwent epithelial-to-mesenchymal transition (EMT) in culture. The translated mesenchymal cells acted as islet progenitors and had a potential to be redifferentiated to insulin-expressing islet-like cell aggregates [16]. Although the EMT process is controversial, many studies have provided evidence that epithelium and mesenchymal stem cells (MSCs) from islets/pancreatic tissues had the ability to differentiate towards *β*-cell phenotype [17, 18]. For nonpancreatic sources, most studies have indicated that the differentiated cells had either a limited insulin-producing ability or lack of response to glucose stimulation of insulin secretion* in vitro*.

It has also been shown that some genetically modified cells failed to express appropriate cell markers such as NKX6-1 or PDX-1 or abnormally coexpress other hormones like glucagon [1, 2, 19]. Recently, Melton's group generated glucose-responsive *β*-cells from human pluripotent stem cells (hPSC) using a scalable differentiation protocol. These stem-cell-derived *β*-cells expressed markers found in mature *β*-cells and secreted quantities of insulin comparable to adult *β*-cells in response to multiple sequential glucose challenges [1]. This approach initially seemed to hold promise for clinical trial; however drawbacks such as teratoma formation and autoimmunity are yet to be addressed [20].

In the present study, we have chosen mature islet cells from adult pig pancreas as source of cells for *β*-cell generation. The pancreatic islet consists of a huge amount of endocrine cells, such as *α*-cells, *β*-cells, and *δ*-cells, besides progenitor cells either coming from EST or initial MSCs. We hypothesize that these endocrine cells and progenitor cells are more likely to reprogram into hormone-producing *β*-cells than other cells from nonpancreas tissues since the latter may retain some characteristics of their embryonic development [21]. Generating hundreds of millions of functional *β*-cells is challenging, and significant research has focused on the redifferentiation of cells to accomplish this goal. A variety of factors affecting *β*-cell renewal have been examined such as serum-free media, betacellulin, exendin-4, shRNA, hepatocyte growth factor, and aggregation of cells [22]. However, in most studies the insulin mRNA levels have been shown to be much lower than the amount in human *β*-cells. Before redifferentiation, islet-derived cells or other progenitors need to be largely increased in numbers by* in vitro* proliferation in order to generate enough quantities of islets for clinical application.

However, islet-derived cells or progenitors easily lose their properties* in vitro*, since it is difficult to find a niche similar to their native microenvironment* in vivo* necessary for survival. The more properties cells lose during culture, the more difficulty for researchers to meet the requirements for redifferentiating the cells back to their native status. In the present studies, we focused on the proliferation of islet cell numbers, their insulin-producing capacity during cell proliferation, and the influence of culture conditions on gene expression, including insulin, PDX-1, somatostatin, glucagon, and NeroD. We found that all gene expressions decreased sharply in culture and that the cells had higher insulin expression in high glucose media than in low glucose media, suggesting that *β*-cell dedifferentiation could be effectively slowed down in a suitable high glucose condition, while it is possible to generate high quality islet aggregates with these noncompletely dedifferentiated *β*-cells.

## 2. Materials and Methods

### 2.1. Separation of Islets from Porcine Pancreases

Pancreatic tissues were harvested from female pigs (*n* = 10) with body weight range 25–30 kg. The tissues were cannulated via the splenic artery and flushed with 15 mL of ice-cold sterile University of Wisconsin solution (UWS). The islet isolation and purification were performed according to a modified procedure described previously [23]. In brief, pancreas was infused with 100 mL fresh enzyme solution, consisting of 1.5 mg/mL collagenase P (11213873001, Roche Applied Science, Indianapolis, IA) and 100 U/mL DNAse (Sigma). After 20 min of enzymatic digestion at 37°C in a water bath, pancreas was subjected to 1 min period mild mechanical disruption and then filtered through a 450 *μ*M mesh strainer. Subsequently, islets were purified by OptiPrep (Cosmo Bio USA, Inc., Carlsbad, CA) density gradient separation.

### 2.2. Cells Dissociation

Immediately after isolation, porcine islets were washed twice with cold phosphate buffered saline (PBS), suspended in fresh enzyme solution as mentioned above, and incubated for 20 min at 37°C. Islets were then dissociated into single cells by pipetting up and down through a 5 mL pipet for 3–5 min and then filtered through a 40 *μ*m Nylon cell strainer. The cells were counted and viability was assessed by trypan blue exclusion test.

### 2.3. Cell Culture

Dissociated cells were seeded on a tissue culture plate (TCP) with a density of 200–300 cells/mm^2^ and cultured in a humidified atmosphere containing 5% carbon dioxide. In this study, two types of basal media were applied, namely, Connaught Medical Research Laboratories Medium (CMRL-1066) (Sigma) and Dulbecco's Modified Eagle Medium (DMEM) (HyClone Laboratories), supplied with 10% fetal bovine serum (FBS) (Sigma) or 20% porcine serum (PS) (Sigma), 1% penicillin-streptomycin (HyClone Laboratories), and 4.0 *μ*M L-glutamine (as shown in Table 1). The medium was first changed after 3 days and then every other day, when cells reached confluence and were subcultured by treating the plates with 0.05% trypsin-EDTA (GIBCO). Cells were reseeded at a density of 50–100 cells/mm^2^ and expanded in TCP. Cells were cultured up to five passages. The population-doubling time (*T*_*d*_ expressed as hours) was calculated according to (1), where *Q*_1_ and *Q*_2_ were the cell numbers at time *t*_1_ and *t*_2_ respectively.

(1) $$\begin{array}{c} T_{d}=\left(\;t_{2}-t_{1}\;\right)*\left[\;\frac{\log2}{\left(\log Q_{2}-\log Q_{1}\right)}\;\right]. \end{array}$$

### 2.4. Immunostaining

Dissociated cells were casted on a glass slide at a spinning rate of 1000 rpm for 3 min. For cultured cells, they were seeded on 8 well-chamber slide. Cells were fixed with 4% paraformaldehyde (PFA) diluted with PBS for 15 min. For porcine pancreas, tissue specimen was fixed in formalin overnight and then dehydrated and paraffin-embedded in a tissue processor (Leica ASP300S, Leica Microsystems, Glattbrugg, Switzerland); 6 *μ*m sections were cut on a microtome (Leica RM2255). Sections were dewaxed in xylol and rehydrated in a descending series of ethanol solution (99%, 95%, 90%, 80%, 70%, and water) prior to the performance of antigen retrieval in citrate buffer 0.01 M pH 6.0. For immunostaining, cell slides/sections were permeabilized in 2.0% Triton X100 for 3 min and blocked for 15 min at room temperature with protein block solution (X0909, Dako). The cell slides/sections were incubated with the first antibody for one hour and with the second antibody for 45 min at room temperature. The nuclei of cells were stained with DAPI mounting solution (H-1200, Vector Labs.). The primary antibodies used were monoclonal anti-insulin (I2018, Sigma) and the secondary antibodies was horse anti-mouse IgG Texas Red (Vector Labs.). The cell slides/sections were examined using Leica fluorescent microscope, model DMI54000B with a QImaging Retiga-2000RV camera.

### 2.5. Quantitative Real-Time Polymerase Chain Reaction (qTR-PCR)

For the qTR-PCR analysis, we used islets pooled from different pig pancreases, which were digested to isolate the cells. The analysis of gene expression was performed on cells from different pigs and representative results were presented. Total RNA was isolated from cells using PerfectPure RNA cultured cell kit (5 Prime Inc., Gaithersburg, MD, USA) according to the manufacturer's protocol. cDNA was prepared using High Capacity cDNA Reverse Transcription kit (Applied Biosystems, Foster City, CA, USA). qRT-PCR was performed in 20 *μ*L reactions in 96-well plates using cDNA samples from 12.5 ng of total RNA. PCR primers used were GAPDH (F: gtcggttgtggatctgacct, R: gtcctcagtgtagcccagga), 18srRNA (F: gtaacccgttgaaccccatt, R: ccatccaatcggtagtagcg), insulin (F: cttcgtgaaccagcacctg, R: cttgggcgtgtagaagaagc), PDX-1 (F: tcccgtggatgaagtctacc, R: cttgttctcctcgggctct), somatostatin (F: gctctctgaacccaaccaga, R: gaaattcttgcagccagctt), glucagon (F: gatcattcccagctccccag, R: gtgttcatcagccactgcac), and NeuroD (F: gagagccccctgactgattg, R: gcccgagaagattgatccgt). RT-PCR was performed with these primers in the presence of Power SYBR Green PCR Master Mix (Applied Biosystems) in an ABI 7300 Real-Time PCR System. Biological replicates were analyzed in triplicate with gene expression normalized to GAPDH or 18sRNA and fold change determined using the ΔΔ*C*_*t*_ method. In order to clearly display the relative differences of gene expression, log⁡(fold changes relative to GAPDH or 18sRNA) was used as *y*-axis in figures; in addition the relative fold changes were marked on reach column of figures. The undetectable level of fluorescence was set at a cycle threshold (*C*_*t*_) of 35.

## 3. Results

### 3.1. Islets Dissociation and Cell Culture

**Figure 1:**
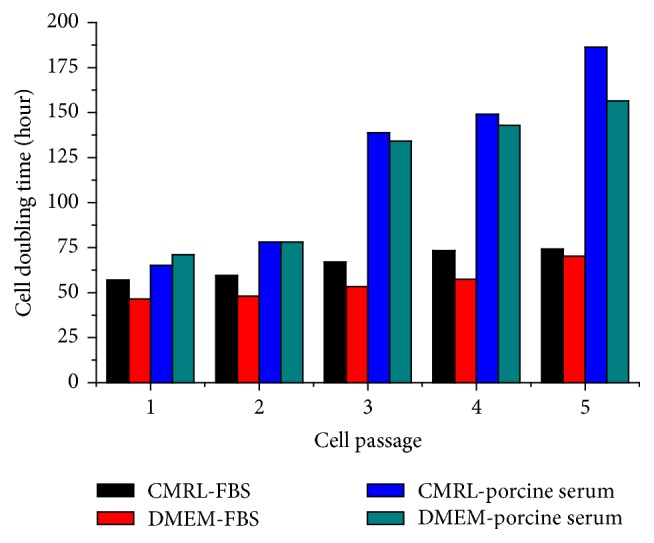
Islet-derived cell proliferation in culture. The doubling time (hours) of islet-derived cells was measured in CMRL and DMEM media supplemented with 10% fetal bovine serum (FBS) and 20% porcine serum (PS) at the indicated passages.

Porcine islets were effectively digested and dissociated into a single cell suspension with very high viability (over 85% viable cells were as determined by trypan blue exclusion dye). We tested the effects of glucose and serum sources (bovine and porcine) on cell proliferation and morphology. The islet-derived cells had a slow attachment rate in tissue culture dishes, and proliferation was observed only 2 days after seeding. The size and morphology of cells changed from a small-size cobblestone-like shape to large, fibroblast-like cells (Supplemental Figure  1 in Supplementary Material available online at http://dx.doi.org/10.1155/2016/5807876) during successive passages (P). Two core media, DMEM and CMRL, were tested for culture of the islet-derived cells, without a significant difference in their effects on cell phenotype and proliferation. However, there was a difference in cell proliferation in media containing 10% FBS compared with 20% porcine serum (PS) (Figure 1). The *T*_*d*_ remained fairly constant in cells cultured with FBS, during 5 passages, around 60 hours. In contrast, *T*_*d*_ value increased sharply in media containing PS, from 65 to 186 hours in P1 and P5, respectively. A high glucose level (4.5 g/L versus 1 g/L) (P5) had no significant effect on cell doubling time.

### 3.2. Expression of Pancreatic Endocrine Genes in Cultured Islet-Derived Cells

**Figure 2:**
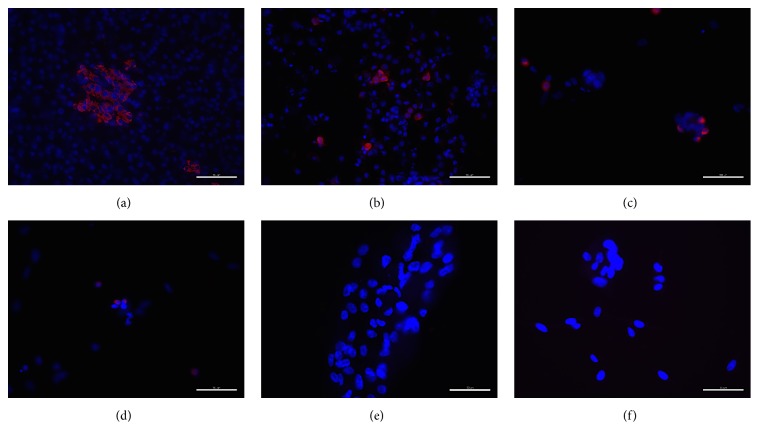
Insulin expression in islet-derived cells in culture. The immunostaining images were obtained from intact pancreatic islet tissue (a), islet-derived cells immediately after islet digestion (b), and after 3 days (c) and 6 (d) days in culture, at 8 days after P1 (e) and at 4 days after P2. Colors show insulin (red) and DAPI (blue) staining. Scale bars = 100 *μ*m (a–d) and 50 *μ*m (e, f).

The dissociated islet cells represent a mixture, including *α*-cells, *β*-cells, *δ*-cells, and stroma cells. Immunostaining for insulin in the cultured cells showed several insulin-positive cells immediately postcell seeding and lower numbers after days 3 and 6 of culture (Figures 2(b)–2(d)). However, insulin-positive cells could not be observed at P1 or P2 (Figures 2(e) and 2(f)). Neither serum source nor glucose level in the media had any effect on these results.

**Figure 3:**
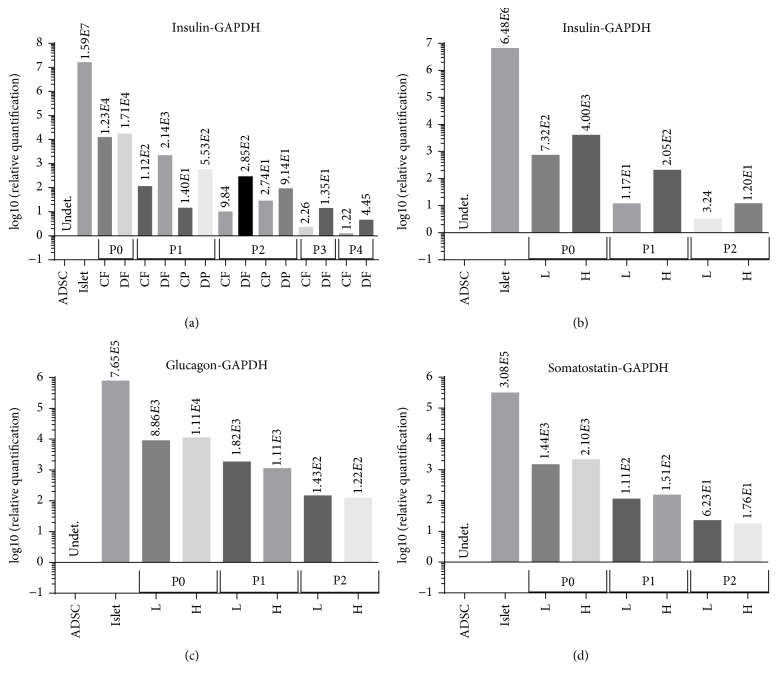
Expression of pancreatic endocrine genes in islet-derived cells cultured with different media. Analysis of gene expression (mRNA) using qRT-PCR was performed on cells isolated from different pigs and representative results are presented. (a) Islet-derived cells were cultured in CMRL media containing 10% FBS (CF) or 20% PS (CP) and in DMEM media containing 10% FBS (DF) or 20% PS (DP) and analyzed for the expression of insulin mRNA. (b–d) Islet-derived cells were cultured in DMEM media containing 10% FBS and 1 g/L (L) or 4.5 g/L (H) glucose and analyzed for the expression of insulin (b), glucagon (c), and somatostatin (d) mRNA. The results are the average fold increase for each indicated mRNA compared with the level of this mRNA in adipose-derived stem cells (ADSC) and normalized for the expression of GAPDH.

**Figure 4:**
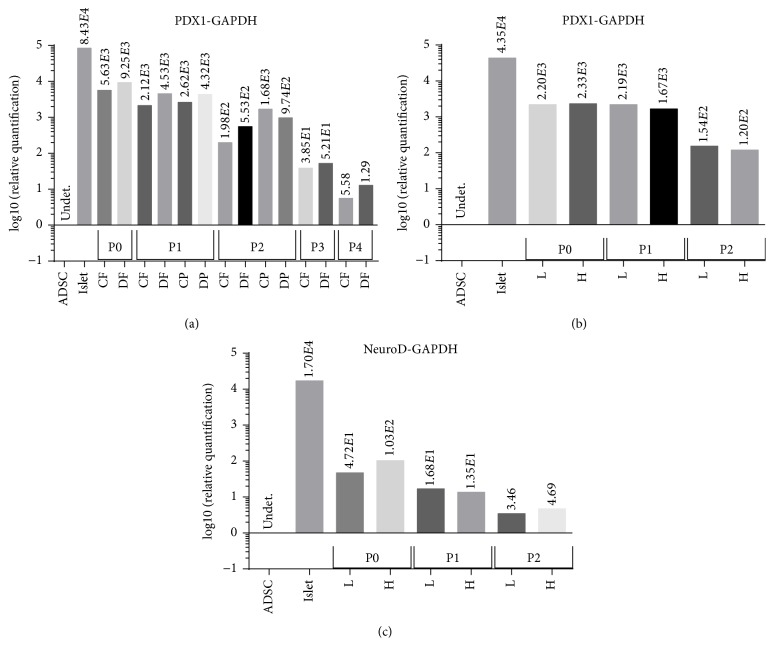
Pdx1 and NeuroD mRNA expression in islet-derived cells cultured with different media. Analysis of gene expression (mRNA) using qRT-PCR was performed on cells isolated from different pigs and representative results are presented. (a) Islet-derived cells were cultured in media as described in Figure 3(a) and analyzed for the expression of Pdx1 mRNA. (b, c) Islet-derived cells were cultured in media as described in Figure 3(b) and analyzed for the expression of Pdx1 (b) and NeuroD (c) mRNA. The results are the average fold increase for each indicated mRNA compared with the level of this mRNA in adipose-derived stem cells (ADSC) and normalized for the expression of GAPDH.

Analysis of gene expression (mRNA) using qRT-PCR was performed on cells isolated from different pigs, and representative results are presented (Figures 3 and 4 and Supplemental Figure  2). Insulin mRNA expression showed a decrease with increasing cell passaging. In general, the levels decreased by about 50–100-fold from P0 to P1 and were further decreased by about 10-fold from P1 to P2, by about 5–20 from P2 to P3 and by about 2-3 from P3 to P4 (Figure 3(a)). These results indicate a dramatic reduction in insulin mRNA when cells were first passaged and a more progressive decrease with subsequent passages. We did not observe a significant difference in insulin mRNA between cells grown in porcine serum and FBS. On the other hand, cells cultured in DMEM core media showed higher insulin mRNA compared with cells cultured in CMRL core media. We have not yet determined the reason for this difference. Similar results were obtained in the analysis of insulin mRNA expression normalized against cellular expression of GAPDH and 18srRNA (Supplemental Figure  2(A)). To test if the islet-derived cells can regulate insulin gene expression in response to increased glucose levels, we analyzed insulin mRNA in media containing low and high glucose, 1 and 4.5 g/L, respectively (Figure 3(b)). We observed a moderate increase in insulin mRNA in the presence of the higher glucose level, but the insulin mRNA levels were greatly reduced upon passaging. These results suggest that the relative portion of *β*-cells in the islet-derived culture greatly decreased over passaging* in vitro*, but they may retain glucose-dependent insulin expression capability.

Since the cultures of islet-derived cells contained a mixture representing the different cell types of the pancreas, we analyzed the expression of glucagon (*α*-cells) and somatostatin (*δ*-cells). Both glucagon and somatostatin mRNA levels decreased by about 10-fold with each passage from P0 to P1 and from P1 to P2 (Figures 3(c) and 3(d)). However, unlike the response of insulin to media glucose, there was no major effect of the glucose level, low and high, on both glucagon and somatostatin mRNA expression.

### 3.3. Expression of Pancreatic Transcription Factors in Cultured Islet-Derived Cells

PDX-1 is considered as a “master regulator” gene of pancreatic cell differentiation and is expressed by both *α*-cells and *β*-cells [2]. We examine Pdx-1 mRNA levels under the different culture conditions, at multiple passages (Figures 4(a) and 4(b)), as described for insulin mRNA. Pdx-1 mRNA levels decreased only by about 2-fold from P0 to P1, compared with the more dramatic decrease in insulin mRNA (Figure 3). The decrease in Pdx1 mRNA was greater between P2 and P3 and between P3 and P4, about 4 and 5, respectively (Figure 4(a)). Similar to insulin mRNA, there was only marginal effects of the serum source, PS and FBS, but unlike insulin, there was only marginal effect of the core media type, DMEM and CMRL, as well as to the glucose levels, low and high. Similar results were obtained when Pdx1 mRNA expression was normalized against cellular expression of 18srRNA (Supplemental Figure  2(A)).

NeuroD is another pancreatic transcription factor involved in the differentiation of pancreatic progenitors to endocrine progenitors [8]. NeuroD mRNA expression in islet-derived cells gradually decreased with cell passage number, by 3–10-fold from P0 to P1 and by about 3-4-fold from P1 to P2, and there was no major effect of the glucose levels, low and high (Figure 4(c)).

## 4. Discussion and Conclusion

In ongoing attempts to generate surrogate glucose-responsive, insulin-secreting islet-like cells for the purpose of achieving a cell-based therapy for both T1D and late-stage T2D, multiple* in vitro* approaches have been honed to increase efficiency and functional maturity of glucose-responsive insulin-secreting islet-like cells [24–30]. ES and iPS cells, while often used as the most popular starting cell populations, have some critical deficiencies. For example, the need for efficient differentiation protocols to induce permanent *β*-cell phenotype* in vivo* and avoid formation has reduced the efficiency of this approach for clinical translation. Trans-differentiation of different somatic cell types, like hepatocytes and exocrine pancreatic cells, has conceptual and pragmatic advantages. Both cell types are developmentally closer to islet endocrine tissue and therefore less likely to require extensive differentiation efforts [21]. In addition, since they are terminally differentiated cells teratoma formation is unlikely. Accordingly, we chose to use islet-derived cells because of the high percentage of endocrine cells in the islets. We examined 2 important aspects of cell proliferation, as means to develop alternatives for insulin-secreting islets* in vitro* cell proliferation rates and expression of pancreatic genes including insulin, glucagon, and somatostatin, as well as the pancreatic transcription factors Pdx1 and NeuroD.

We chose CRML-1066 and DMEM as the basal media, supplied with 10% FBS or 20% PS. *T*_*d*_ of cells were increased with passaging, consistent with the results of Kayali et al. [15]. Although we found that media supplemented with 20% PS were more compatible with porcine cells, our results also showed that cells cultured in media supplemented with 10% FBS had a lower *T*_*d*_. The exact reason for this observation is unclear but could potentially be due to excessive levels of growth factors present in the fetal serum. We observed a dramatic decrease in the numbers of insulin-positive cells and in insulin mRNA expression during time in culture, similar to results reported for human islet cells [31] and by Kayali et al. [15]. In addition, our results also showed a similar decrease in glucagon and somatostatin mRNA expression. On the other hand, the decrease in Pdx1 and NeuroD mRNA expression was slower than that of insulin, even after 2 consecutive passages. Together, these results suggest that although we observed a rapid loss of function in dispersed islet cells in culture, the islet-derived cells may maintain their pancreatic “identity” by extended expression of pancreatic transcription factors.

Glucose homeostasis in mammals requires the proper regulation of insulin secretion from pancreatic islets [32]. *β*-Cells respond to increased glucose levels through calcium signaling, leading to membrane depolarization and an influx of calcium ions, which triggers insulin exocytosis [1]. High glucose concentrations cause trans-location of Pdx1 from the nuclear periphery to the nucleoplasm with a concomitant increase in insulin gene transcription [32, 33]. *β*-Cells are normally maintained within a very narrow range of glucose concentrations about 0.65–1 g/L, but when forced into an environment of even mild hyperglycemia their phenotype and function change [34]. Some previous studies have used high glucose levels to induce cell differentiation towards *β*-cell and stimulate expression and release of insulin. For example hepatocytes could be effectively differentiated into endocrine cell phenotypes in serum-free medium with high glucose (4.5 g/L) and activin A, and the differentiated cells were shown to contain C-peptide and release insulin in response to physiological glucose levels [35]. Also, Racanicchi et al. characterized neonatal pig liver-derived progenitors and evaluated their trans-differentiation capacity into pancreatic endocrine-like cells and showed that a high glucose concentration in the media (2.7 g/L) improved the differentiation process [36]. Similarly, Russ et al. demonstrated that dedifferentiated *β*-cells could be redifferentiated by stimulation with high glucose level (4.5 g/L) in the medium for 2 days during which the expression of insulin, Pdx1, and MAFA significantly increased over time [37]. In the present study, only insulin expression was significantly affected by glucose level. We found that insulin expression was about 4- to 18-fold higher in high glucose media compared with low glucose media during 3 consecutive passages, suggesting that the cells maintained their ability to “sense” glucose and upregulate insulin expression.

In the present study we hypothesized that dedifferentiated islet-derived cells may be a good source for generating insulin-secreting cells because they will likely be easier to redifferentiate back to *β*-cells phenotype. Our results indicate that, although the expression of functional genes such as insulin glucagon and somatostatin decreased sharply during cell passaging, expression of pancreatic transcription factors such as Pdx1 and NeuroD was maintained during long-term culture. These results suggest that the islet-derived cells are potentially suitable for redifferentiation protocols to reacquire a *β*-cell phenotype. Although more research is needed, these early results are encouraging for the future *β*-cell therapy in diabetes.

## Supplementary Material

Supplemental figure 1 shows Islet-derived cell shape in culture. Images of Islet-derived cells were obtained at the indicated passage numbers and the duration (days) after each passage. The cells were cultured in CMRL media containing with 10% FBS (a) or 20% porcine serum (c) and in DMEM media containing 10%FBS (b) with 20% porcine serum (d). Cell size and morphology changed from small-size cobblestone-like shape into big-size fibroblast-like cells during the 5 passages. Supplemental figure 2 Expression of insulin and PDX-1 mRNA expression in islet-derived cells cultured with different media and normalized for 18sRNA expression. Islet derived cells were cultured in media as described in Fig 3A. Analysis of insulin (A) and Pdx1 (B) mRNA expression using qRT-PCR was performed on cells isolated from different pigs and representative results were presented. The results are the average fold increase for each indicated mRNA compared with the level of this mRNA in adipose-derived stem cells (ADSC), and normalized for the expression of 18sRNA.

## Figures and Tables

**Table 1:** The information of media used for cell culture.

	Basal medium	Serum	Glucose (g/L)
CMRL-F	CMRL-1066	10% FBS	1.0
CMRL-P	CMRL-1066	20% PS	1.0
DMEM-HG-F	DMEM	10% FBS	4.5
DMEM-HG-P	DMEM	20% PS	4.5
DMEM-LG-F	DMEM	10% FBS	1.0
