# Supplementary material for: In Vitro Proliferation of Porcine Pancreatic Islet Cells for β-Cell Therapy Applications
Source: J Diabetes Res. 2016 Dec 6;2016:5807876. doi: 10.1155/2016/5807876 (PMC5168554; doi:10.1155/2016/5807876)
Supplement: Supplementary file 1 — Supplemental figure 1 shows Islet-derived cell shape in culture. Images of Islet-derived cells were obtained at the indicated passage numbers and the duration (days) after each passage. The cells were cultured in CMRL media containing with 10% FBS (a) or 20% porcine serum (c) and in DMEM media containing 10%FBS (b) with 20% porcine serum (d). Cell size and morphology changed from small-size cobblestone-like shape into big-size fibroblast-like cells during the 5 passages. Supplemental figure 2 Expression of insulin and PDX-1 mRNA expression in islet-derived cells cultured with different media and normalized for 18sRNA expression. Islet derived cells were cultured in media as described in Fig 3A. Analysis of insulin (A) and Pdx1 (B) mRNA expression using qRT-PCR was performed on cells isolated from different pigs and representative results were presented. The results are the average fold increase for each indicated mRNA compared with the level of this mRNA in adipose-derived stem cells (ADSC), and normalized for the expression of 18sRNA. [file 5807876.f1.pdf]

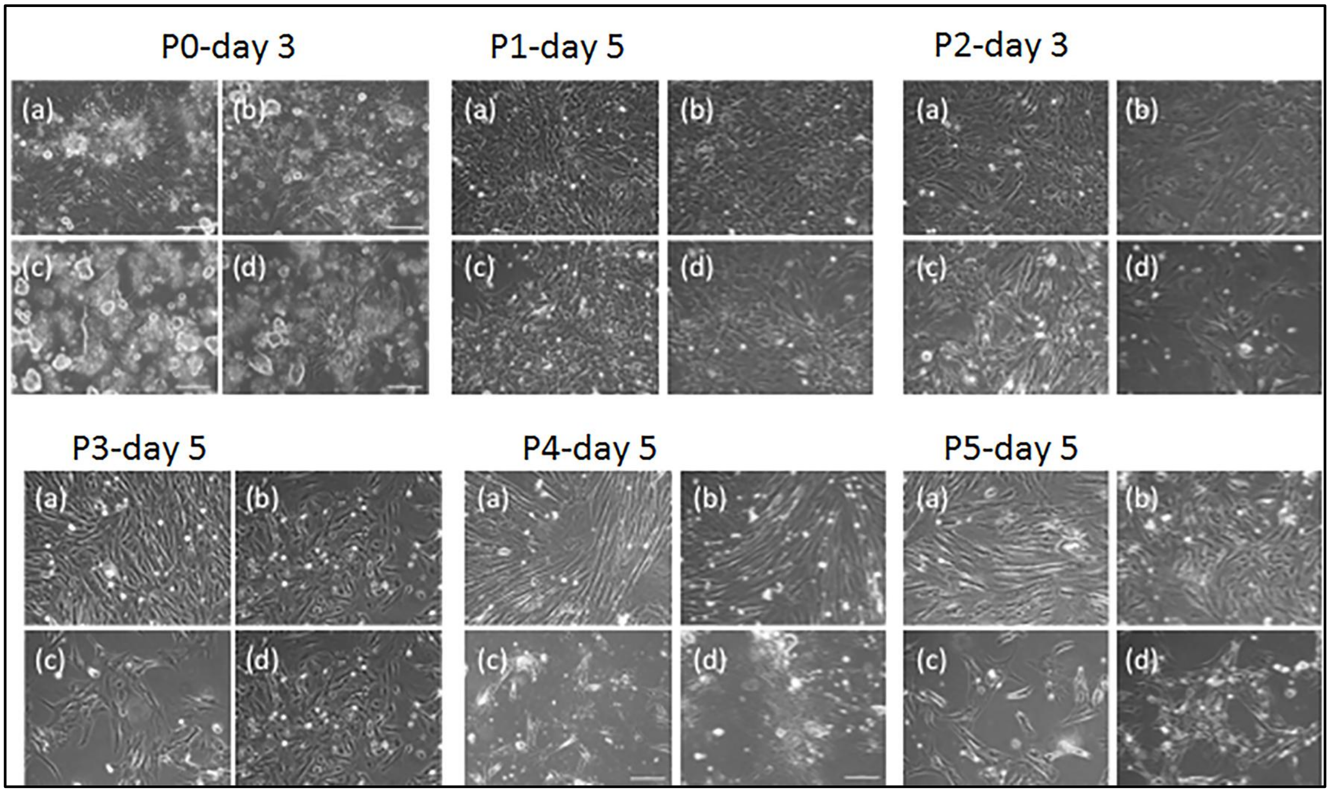

Supplemental Fig. 1 Islet-derived cell shape in culture. Images of Islet-derived cells were obtained at the indicated passage numbers and the duration (days) after each passage. The cells were cultured in CMRL media containing with 10% FBS (a) or 20% porcine serum (c) and in DMEM media containing 10%FBS (b) with 20% porcine serum (d). Cell size and morphology changed from small-size cobblestone-like shape into big-size fibroblast-like cells during the 5 passages.

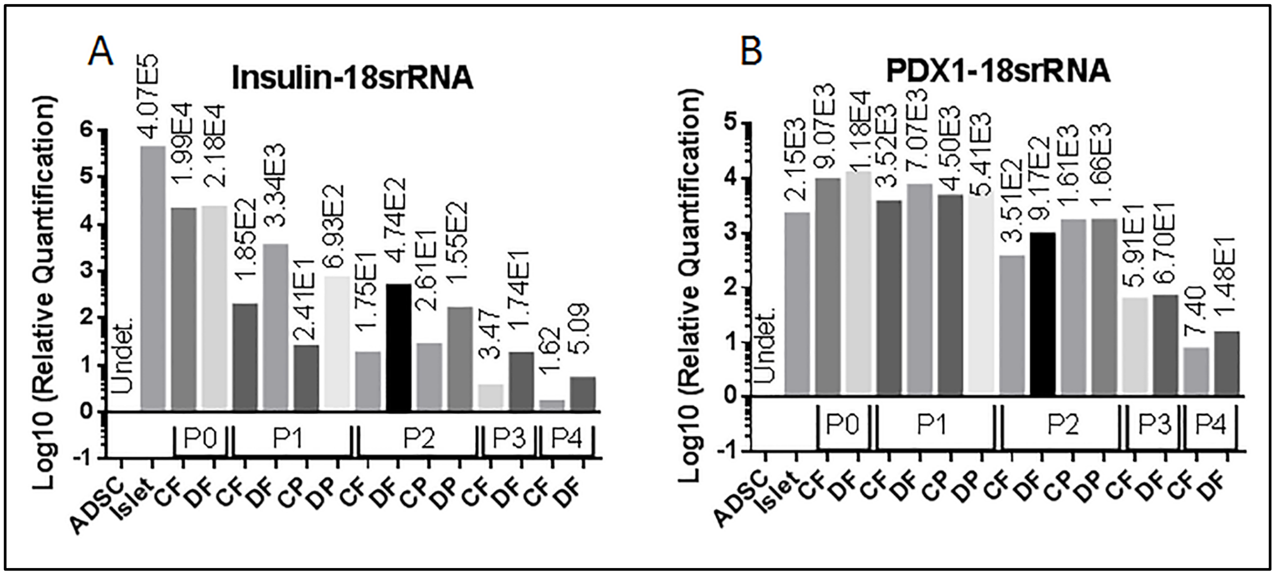

Supplemental Fig. 2 Expression of insulin and PDX-1 mRNA expression in islet-derived cells cultured with different media and normalized for 18sRNA expression. Islet derived cells were cultured in media as described in Fig 3A. Analysis of insulin (A) and Pdx1 (B) mRNA expression using qRT-PCR was performed on cells isolated from different pigs and representative results were presented. The results are the average fold increase for each indicated mRNA compared with the level of this mRNA in adipose-derived stem cells (ADSC), and normalized for the expression of 18sRNA.
